# 1834. Impact of COVID-19 and Socioeconomic Status on Food Insecurity in the US Adult Population: Analysis of the 2021 National Health Interview Survey

**DOI:** 10.1093/ofid/ofad500.1663

**Published:** 2023-11-27

**Authors:** Jincong Q Freeman, Xinyi Li, Yong Gun Lee, Adam W Scott, Yijin Xiang

**Affiliations:** The University of Chicago, Chicago, Illinois; Milken Institute School of Public Health, The George Washington University, Washington, District of Columbia; Rutgers, The State University of New Jersey, New Brunswick, New Jersey; University of Minnesota Medical School, Minneapolis, Minnesota; Emory Univeristy, Atlanta, Georgia

## Abstract

**Background:**

Food insecurity (FI) is a public health concern and negatively affects the health and well-being of many adults in the US, which may have been exacerbated since the COVID-19 pandemic. However, the relationship between COVID-19 and FI among US adults has not been elucidated.

**Methods:**

Data were from the 2021 National Health Interview Survey that used stratified clustering sampling to interview US adults aged ≥ 18 years. COVID-19 infection (yes/no) was per self-report. FI, determined by a 10-item questionnaire assessing household food situations in the past 30 days, was dichotomized (yes/no). Unweighted frequencies and weighted percentages were calculated and compared using Rao-Scott chi-square tests. Multivariable logistic regression was used to examine differences in FI prevalence by COVID-19. All analyses accounted for complex survey design and weights.

**Results:**

The unweighted sample size was 28,274 (mean age 48.3 years). Overall, 13.5% (95% CI: 13.0%-14.1%) had COVID-19, and 5.9% (95% CI: 5.5%-6.3%) experienced FI. Adults with COVID-19 reported a significantly higher percentage of FI than those without (7.3% [95% CI: 6.1%-8.6%] vs. 5.6% [95% CI: 5.2%-6.0%], *p*=0.003). Most (63.2%) were White, followed by 16.7% Hispanic, 11.5% Black, 5.9% Asian, and 2.6% other races; 22.6% were on Medicaid/Medicare and 9.8% were uninsured; 12.9% received food stamps in the past 12 months. In the adjusted model, adults with COVID-19 were more likely to have experienced FI than those without (adjusted odds ratio [aOR]=1.29, 95% CI: 1.07-1.56). Black (aOR=2.08, 95% CI: 1.72-2.50) or Hispanic (aOR=1.26, 95% CI: 1.03-1.53) adults were more likely than their White counterparts to have experienced FI. Uninsured adults (aOR=2.67, 95% CI: 2.15-3.31) or those on Medicaid/Medicare (aOR=1.94, 95% CI: 1.60-2.35) had greater odds of having experienced FI than those privately insured. Adults receiving food stamps were more likely to have experienced FI than those not receiving (aOR=2.87, 95% CI: 2.36-3.50).

**Figure:**
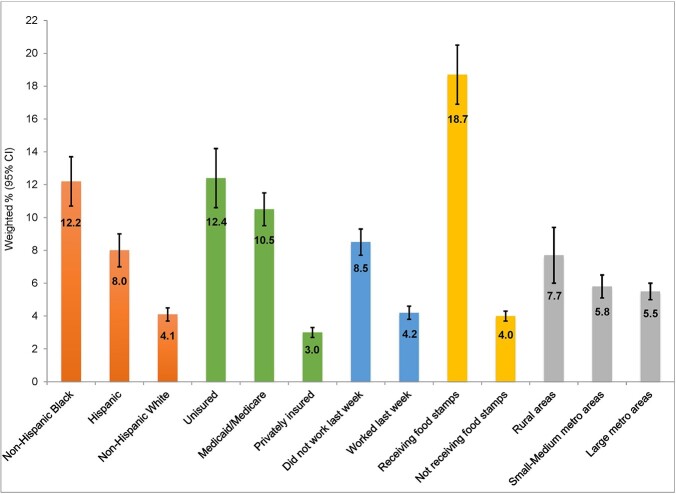

**Conclusion:**

In this study of a nationally representative sample, our findings highlight a higher prevalence of FI among adults with COVID-19, with lower socioeconomic status, or in racial/ethnic minority communities and suggest the need for intervention strategies addressing FI in these adult populations.

**Disclosures:**

**All Authors**: No reported disclosures

